# A case of severe traumatic cardiac injury due to a leadless pacemaker that required surgical repair

**DOI:** 10.1093/omcr/omab146

**Published:** 2022-01-24

**Authors:** Motoaki Higuchi, Kisato Mitomi, Yoshiro Chiba

**Affiliations:** Department of Cardiology, Mito Saiseikai General Hospital, 3-3-10 Futabadai, Mito, Ibaraki Prefecture 311-4145, Japan; Department of Cardiovascular Surgery, University of Tsukuba Hospital, 2-4-1 Amakubo, Tsukuba, Ibaraki Prefecture 305-8576, Japan; Department of Cardiology, Mito Saiseikai General Hospital, 3-3-10 Futabadai, Mito, Ibaraki Prefecture 311-4145, Japan

## TEXT

**Figure 1:**
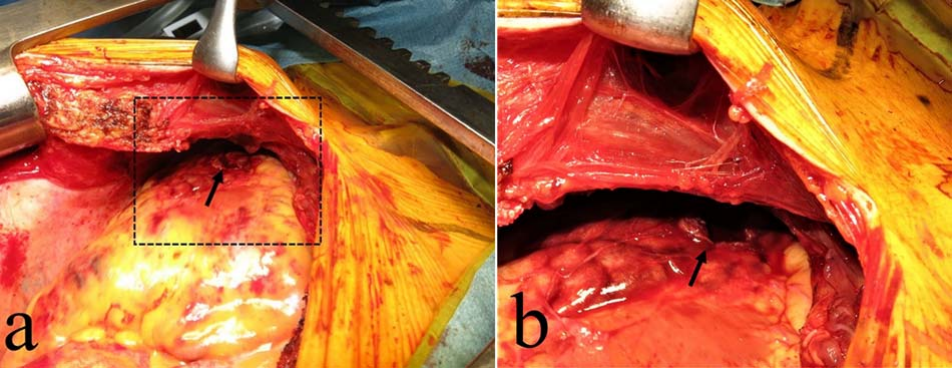
thoracotomy findings. (**a**) The left side of the figure is the head side. The arrow indicates the site of traumatic cardiac injury. (**b**) An enlarged image of the part surrounded in (a).

A 96-year-old woman with heart failure due to a 2:1 atrioventricular block was hospitalized. A Leadless pacemaker, Micra-transcatheter pacing system (Micra-TPS) (Medtronic, Minneapolis, MN), was implanted through the right femoral vein. The pacemaker had a poor threshold after the first deployment and was retrieved. It was redeployed, and a good threshold was achieved. Afterward, her systolic blood pressure decreased from 180 mmHg (at the time of admission) to 70 mmHg, resulting in cardiogenic shock. Echocardiography showed pericardial effusion, which was suggestive of cardiac injury and cardiac tamponade. She underwent emergency pericardial drainage and 300 ml of bloody pericardial fluid was drained, after which she recovered from shock. However, due to persistent drainage of bloody pericardial fluid, we performed an emergency thoracotomy. We found a full-thickness traumatic myocardial injury measuring ~10 mm on the anterior surface of the right ventricle (Fig. 1a and b). The damaged part was not at the site where the Micra pacemaker was implanted, but the damage had occurred during the initial deployment. She underwent cardiac repair at the same site and became hemodynamically stable. She was discharged 2 weeks postoperatively.

Micra-TPS is expected to reduce the risk of device infections and lead complications compared with conventional intravenous pacemakers [1]. However, the Micra-TPS implantation method has been reported to have a 1.6% risk of myocardial injury [2], as it involves pressing a dedicated 23 Fr (~8 mm) delivery system against the right ventricular wall in order to deploy it. This is an important, life-threatening complication of Micra-TPS implantation. Therefore, surgical implantation of a Micra-TPS should be done with caution. Myocardial injury caused by Micra-TPS causes major myocardial damage, and conservative treatment with pericardial drainage may be difficult; hence, surgical repair should be considered immediately.
